# Consistency of arsenic speciation in global tobacco products with implications for health and regulation

**DOI:** 10.1186/s12971-014-0024-5

**Published:** 2014-12-11

**Authors:** Robert CJ Campbell, W Edryd Stephens, Andrew A Meharg

**Affiliations:** Department of Earth & Environmental Sciences, University of St Andrews, Irvine Building, North Street, St Andrews, Fife KY16 9AL UK; Institute for Global Food Security, Queen’s University Belfast, David Keir Building, Stanmills Road, Belfast, BT9 5AG UK

**Keywords:** Arsenic, Speciation, Tobacco, Smoking, Health, Regulation

## Abstract

**Background:**

Tobacco smoke is a major risk to the health of its users and arsenic is among the components of smoke present at concentrations of toxicological concern. There are significant variations in human toxicity between inorganic and organic arsenic species and the aim of this study was to determine whether there are predictable relationships among major arsenic species in tobacco that could be useful for risk assessment.

**Methods:**

14 samples of tobacco were studied spanning a wide range of concentrations in samples from different geographical regions, including certified reference materials and cigarette products. Inorganic and major organic arsenic species were extracted from powdered tobacco samples by nitric acid using microwave digestion. Concentrations of arsenic species in these extracts were determined using HPLC-ICPMS.

**Results:**

The concentrations of total inorganic arsenic species range from 144 to 3914 μg kg^-1^, while organic species dimethylarsinic acid (DMA) ranges from 21 to 176 μg As kg^-1^, and monomethylarsonic acid (MA) ranges from 30 to 116 μg kg^-1^. The percentage of species eluted compared to the total arsenic extracted ranges from 11.1 to 36.8% suggesting that some As species (possibly macro-molecules, strongly complexed or in organic forms) do not elute from the column. This low percentage of column-speciated arsenic is indicative that more complex forms of arsenic exist in the tobacco. All the analysed species correlate positively with total arsenic concentration over the whole compositional range and regression analysis indicates a consistent ratio of about 4:1 in favour of inorganic arsenic compared with MA + DMA.

**Conclusions:**

The dominance of inorganic arsenic species among those components analysed is a marked feature of the diverse range of tobaccos selected for study. Such consistency is important in the context of a WHO expert panel recommendation to regulate tobacco crops and products using total arsenic concentration. If implemented more research would be required to develop models that accurately predict the smoker’s exposure to reduced inorganic arsenic species on the basis of leaf or product concentration and product design features.

## Background

Among several thousand chemical compounds documented in tobacco smoke 98 have published inhalation risk factors, and 11 of these are metals or metalloids [1]. The World Health Organisation (WHO) expert panel on tobacco regulation (TobReg) recently reviewed the published literature on metals and metalloids in tobacco and smoke, concluding that arsenic (As), cadmium (Cd), nickel (Ni) and lead (Pb) are of sufficient concern that they should be subject to regulation [2]. The panel recommended that “manufacturers … test cured tobacco … for levels of arsenic, cadmium, lead and nickel”. Underlying this recommendation is the assumption that the concentration of a metal or metalloid in smoke has a predictable relationship to its concentration in the precursor tobacco. Another dimension of the hazard is speciation, the chemical form and oxidation state of a metal or metalloid in tobacco and smoke. Speciation strongly influences bioavailability, reactivity with cellular materials and detoxification mechanisms, and ultimately toxicity.

This paper focuses on arsenic, one of the four metals and metalloids currently proposed for regulation [2] and addresses the variability in chemical speciation of arsenic in 14 tobacco products sampled from a wide range of geographical localities and As concentrations. Studies of the risks associated with dietary exposure to arsenic tend to emphasise two aspects of its speciation, namely the chemical species (primarily whether present as organic and/or inorganic compounds) and oxidation state (As(III) or (As(V), or some combination). There is now a large body of evidence that implicates inorganic As(III) species in human toxicity associated with exposure to arsenic in the gastrointestinal tract, however the variable risks of exposure to arsenic species during inhalation are less well characterised [3,4]. Nevertheless long-term, low-dose exposure to inorganic arsenic by whichever pathway is implicated in increased mutagenesis [3] and habitual smoking may involve decades of low-dose exposure. Estimates of the fraction of total arsenic released into smoke during tobacco combustion falls in the range 9-16% [5] suggesting that hundreds of ng of As per cigarette could be transferred although measured quantities in machine smoking using low arsenic products indicate that transference ranges from <1 ng to about 70 ng per cigarette [6-9]. It should be noted that tobacco is not the only determinant of arsenic in smoke, cigarette design (tobacco mass, cigarette length, packing, filter ventilation, etc.) also has a major influence on transference [9]. Supporting the calls for arsenic regulation are the recent findings that arsenic in smoke condensate is present primarily in the As(III) oxidation state [10] largely as inorganic species [11], i.e. potentially the most toxic form. Notwithstanding these findings, the inter-relationships between arsenic concentrations and the species makeup in both tobacco and smoke are not yet established.

Various approaches to the speciation of arsenic in tobacco and smoke have been published recently, utilising HPLC-ICPMS and synchrotron XANES methods [10-13]. These authors established 89% of the total water-soluble arsenic in US Reference Cigarette 3R4F is inorganic, dominantly in the As(V) oxidation state, with indications of minor quantities of less toxic organic species including dimethylarsinic acid (DMA) and monomethylarsonic acid (MA) [11,12] with Zhang et al. also identifying significant quantities of arsenobetaine and arsenocholine [13]. They also demonstrated that arsenic transported in smoke takes the form of more toxic As((III) species, probably as the result of combustion-related reduction [10].

It is not known whether these results can be extrapolated to global cigarette tobaccos and here we address this question using tobaccos chosen to represent the typical range of arsenic concentrations sampled from diverse global localities and analysed using a HPLC-ICPMS. The goal is to quantify any predicable relationships among the arsenic species for incorpatoration in quantitative risk assessment of different tobacco products.

## Methods

### Samples

14 tobacco samples were selected for study including reference materials, authentic commercial US, UK and Chinese cigarette brands, and counterfeit cigarettes seized by UK Customs chosen for their elevated levels of As [14]. These samples and their geographical locations (where known) and concentration ranges are shown in Table 1.

**Table 1:** **Samples and results**

**Sample**		**Total**		**Species**							**Extracts**		**Extract efficiency %**
	**Rationale for selection**	**As**		**DMA**		**MA**		**Organic As**		**Σ As spec**	**As**		
		**μg kg** ^**-1**^	**n**	**μg kg-** ^**1**^	**n**	**μg kg** ^**-1**^	**n**	**μg kg** ^**-1**^	**n**	**μg kg** ^**-1**^	**μg kg** ^**-1**^	**n**	
**Reference**													
CTA OTL-1	CRM Oriental Tobacco Leaf (Bulgaria)	611 ± 41	2	31 ± 1	2	<LoD	1	127 ± 8	2	158	304 ± 3	2	25.9
CTA VTL-2	CRM Virginia Tobacco Leaf (Bulgaria)	1008 ± 39	2	54 ± 6	2	<LoD	1	256 ± 17	2	310	569 ± 59	2	30.8
1R4F	Research Cigarette typical of US low tar blends	465 ± 3	2	24 ± 4	2	<LoD	1	96 ± 2	2	120	209 ± 3	2	25.8
1R5F	Research Cigarette typical of US ultra-low tar blends	318 ± 10	2	37 ± 5	2	<LoD	1	78 ± 26	2	115	154 ± 8	2	36.2
GBW 08514	Chinese tobacco standard	656	1	26	1	30	1	112	1	168	310	1	25.6
GBW 08515	Chinese tobacco standard	429	1	23	1	<LoD	1	62	1	85	191	1	19.8
**Samples**													
B1	Major US brand	443	1	39	1	<LoD	1	79	1	118	192	1	26.6
B2	Major UK brand	191	1	23	1	<LoD	1	39	1	62	113	1	32.5
B3	Major UK brand	317	1	21	1	<LoD	1	66	1	87	162	1	27.4
B4	Major UK brand	144	1	<LoD	1	<LoD	1	16	1	16	82	1	11.1
B5	Major Chinese brand	816	1	49	1	33	1	218	1	300	409	1	36.8
B6	Counterfeit with unusually high arsenic concentration	3914 ± 90	3	150 ± 15	4	45 ± 12	4	948 ± 36	4	1143	1791 ± 47	4	29.2
B7	Counterfeit with unusually high arsenic concentration	3504	1	176	1	116	1	846	1	1138	1777	1	32.5
B8	Counterfeit with high arsenic concentration	2339	1	120	1	42	1	487	1	649	1180	1	27.7

Reference materials and samples selected for As species determination with corresponding sample codes and comments on the rationale for selection. Certified values are available for the CTA standards [15,16], information values provided for other samples were determined for this study by X-ray fluorescence spectrometry [17]. Concentrations of total As, DMA, MA, inorganic As, sum of extracted As species and extract total As in six reference tobacco and eight cigarette tobacco samples, with extraction efficiencies (∑ As sp./Totals) and column recovery (∑ As sp./Extracts). The limit of detection (LoD) for MA is 39 μg kg^-1^.

### Quality control and assurance

CTA-OTL-1 and CTA-VTL-2 (Institute of Nuclear Chemistry and Technology, Warsaw, Poland) are certified reference materials of powdered tobacco for trace element concentrations in Oriental and Virginia tobacco leaves respectively [15-17]. GBW08514 and GBW08515 (National Institute of Metrology, Beijing, China) are tobacco reference standards for some trace elements (not including As) in tobacco [18] while 1R4F and 1R5F (University of Kentucky, Lexington, USA) are reference cigarettes typical of US low tar and ultra-low tar products used for smoking experiments. 1R4F has 9.9 ± 0.4 mg cig^-1^ tar and 0.76 ± 0.03 mg cig^-1^ nicotine and 1R5F has 3.3 ± 0.4 mg cig^-1^ tar and 0.31 ± 0.1 mg cig^-1^ of nicotine) [19]. While only two of these reference tobaccos have certified values for As concentration all six were selected for study as they represent homogenous powder and tobacco samples of different provenance which are easily obtainable by other laboratories. No certified reference materials for As speciation in plant materials had been formally validated at the time the analyses were conducted.

Blanks and a single sample were analysed in triplicate, and the reference materials were analysed in duplicate for elemental concentrations and species extraction. A standard mix of As(III), As(V) (both Sigma, St Louis, USA), DMA and MA (both Argus Chemicals, Vernio, Italy) in concentrations of 5 μg l^-1^ was prepared for calibration of the elution sequence.

### Elemental and extract arsenic concentrations

Sample preparation followed a modification of an established procedure [20]. Sample (0.250 g) was weighed into Teflon vessels (DAP-80s, Berghof GmbH, DE), with 10.0 ml nitric acid (70% v/v) and the reaction accelerated in a pressure- and heat-controlled microwave digestion system (Speedwave MWS-3+, Berghof, DE) programmed to ramp temperature from 120 to 170°C over a 55-min cycle, pressure limited to 30 bar. Samples were then diluted to 0.250 l (a total dilution factor of 1000) with double de-ionised water (Q-gard 1 Gradient A10, Millipore, FR). An aliquot of 5.0 ml of the sample was pipetted into disposable ICP-MS vials together with 5.0 ml of an internal standard solution containing 25 μg l^-1^ germanium (Ge), 5 μg l^-1^ indium (In) and 50 μg l^-1^ rhenium (Re) (ICP-MS single element standards, Inorganic Ventures Inc., Christiansburg, USA). Analysis was performed using an ICP-MS (X-Series 2, Thermo Scientific Corp., UK) quadrupole mass spectrometer with collision cell technology using kinetic energy discrimination (CCTED) to determine As (75 m/z), Ge (72 m/z), In (115 m/z) and Re (185 m/z).

### Arsenic species concentrations

Species extraction sample preparation involved weighing 0.200 g of sample for digestion over 24 hours in 10.0 ml nitric acid (1% v/v) in 50.0-ml centrifuge tubes (a dilution factor of 50). A microwave reaction accelerator system (MARS CEM, Matthews Inc., US) was utilised, programmed to ramp temperature from 55 to 95°C over a 65-min cycle [21]. Samples were then placed in a freezer at -20°C to limit the extent of transformation between species. 24 hours before analysis samples were removed from the freezer and allowed to reach room temperature. Samples were then centrifuged at 15 kg for 10 min, and 0.50 ml of supernatant was pipetted into HPLC-ICP-MS vials with 0.050 ml hydrogen peroxide (H_2_O_2_, Aristar, VWR, Leuven, Belgium) for the analysis. Analysis was performed using HPLC (Agilent 1100 series, Agilent Technologies Inc., DE) fitted with an anion-exchange column (250 by 4.6 mm PRP-X100 10 μm, Hamilton Company, CH & US) [21]) with a buffer solution consisting of 6.66 mM ammonium hydrophosphate (NH_4_H_2_PO_4_) and 6.66 mM ammonium nitrate (NH_4_NO_3_) (AnalaR from BHD chemicals Ltd., Poole, England), adjusted to a pH of 6.2 using ammonia [22], that was connected post-column to an ICP-MS (Agilent 7500) [23] via a Teflon t-piece, directly to the nebulizer to determine As, Rh (103 m/z) and Se (77 and 82 m/z).

Terminology and definitions for chemical species follow established practice [24,25].

### Data analysis

Statistical tests were performed in Minitab 14 (Minitab Inc., US). All data were tested for normality using the Anderson-Darling method. Data that did not conform were transformed with power 10, natural and natural gamma logarithms, square root, sine (angle in radians) or, if necessary, Box-Cox. Relationships between the arsenic species were established using correlation and regression analysis.

## Results

### Total arsenic concentrations

Analytical recoveries for total As in the CRMs CTA-OTL-1 (certified values = 539 ± 60 μg As kg^-1^) and CTA-VTL-2 (certified value = 969 ± 62 μg As kg^-1^) were 113 ± 7% and 104 ± 4%, respectively (n = 2) (Table 1). The limit of detection for total As by ICP-MS was 21 μg kg^-1^ determined by mean plus three standard deviations of the blanks (n = 3). There are no certified values for As in reference tobaccos GBW 08514 and GBW 05815, and reference cigarettes 1R4F and 1R5F. Accuracy of the total arsenic determinations is indicated by good agreement with certified values of the reference standards CTA-OTL-1 and CTA-VTL-2 supported by good precision indicated by low standard deviations for these standards (Table 1).

Total arsenic in these 14 samples ranges from 144 to 3914 μg As kg^-1^ (median = 538; n = 14), with total As concentrations in extracts ranging from 82 to 1791 μg As kg^-1^ (median = 257; n = 14) (Table 1).

As concentrations in the reference standards and legal samples of this study range from 144 - 1008 μg kg^-1^ (median = 443; n = 11) and are similar to those in the literature [14,26], indicating that the tobacco plants were probably cultivated in conditions largely uncontaminated with As (Table 1). In contrast the plants used to make the three illicit (counterfeit) products have much higher As concentrations (2339 – 3914 μg As kg^-1^; median = 3504, n = 3) and were probably cultivated on soils quite heavily contaminated with arsenic due to natural enrichments in the soil, addition of contaminated fertilisers such as sewage sludge, and/or treatment with arsenical pesticides.

### Arsenic speciation

**Figure 1:**
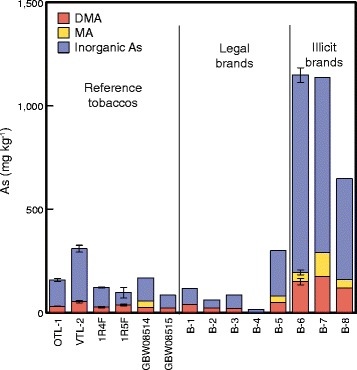
**Species concentrations.** Concentrations of DMA, MA and inorganic arsenic in six reference tobaccos and eight tobacco products (legal and illicit). Error bars set at one standard deviation.

DMA concentrations ranged from 21 to 176 μg kg-1 (median = 37 μg kg-1; n = 13), MA from <39 μg kg^-1^ (limit of detection) to 116 μg kg-1 (median = 42 μg kg-1; n = 5), and inorganic As from 16 to 948 μg kg-1, (median = 104 μg kg-1; n = 14) (Table 1, Figure 1). Across all samples, the proportion of extractable as elutable As species ranged from 11.1 to 36.8% (Table 1), indicative of values expected from highly fermented complex organic matrices such as tobacco leaf. Column recoveries ranged from 19.5 to 74.7%, again reflecting that yet unknown, and potentially macromolecule incorporated, As species remain unextracted. It is relevant here that a study of As speciation in Chinese tobacco also identified significant quantities of arsenobetaine and arsenocholine [13].

Estimating species concentrations using chemical extraction methods suffers from potential inaccuracy due to incomplete extraction recoveries, and this and the other studies were hampered by the lack of an accepted speciation reference standard for As in plant material. Notwithstanding, the specific analytical procedure used in this study has been validated and compares favourably with other extraction techniques [27]. Another technical difficulty involving the calculation of species concentrations from the spectra, due to the coelution of the As(III) and DMA elution peaks, was overcome by oxidizing As(III) to As(V) by the addition of H_2_O_2_ (Aristar, VWR Aristar, Leuven, Belgium) [21,22]. The addition of sufficient to excess H_2_O_2_ converts all inorganic As(III) in a sample to As(V) with no degradation of organic arsenicals MA and DMA [28]. An earlier study of the potential to decompose organoarsenic species during this extraction step found that organic arsenic species are very resistant to attack under even harsher extraction conditions than used in the present study [29].

As(III) elutes at the ejection front, and As(V) much later in a distinct peak; oxidisation also enables inorganic As (As(III) and As(V)) to be differentiated from cationic species which, if present, would also elute with the solvent front [30]. Broadly similar results for inorganic As were obtained for 1R4F (this study, Table 1 and Figure 1) and 3R4F made with a similar blend of tobacco types and analysed in an independent study [12].

These data demonstrate that MA and DMA are present in minor concentrations compared with inorganic As species in all 14 tobacco samples, as shown graphically in Figure 1, extending the earlier finding on 3R4F [12] to a much wider range of reference tobaccos and publicly-consumed products. Also notable is that the same pattern is found in samples of counterfeit products previously shown to have high arsenic concentrations [14] (B6-B8 in Figure 1). Total arsenic in these samples varies by a factor of over 20 yet the ratios of inorganic:DMA:MA concentrations remain relatively consistent.

While As was found to be present principally in inorganic form with minor DMA and MA contributions, As-thiol complexes may represent a significant proportion of the species not extracted or detected in this analysis [31].

## Discussion

### Relationships between arsenic species

**Figure 2:**
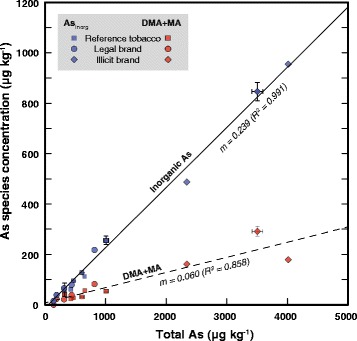
**Regression analysis.** Regressions analysis of extractable inorganic arsenic and DMA + MA against total arsenic in six reference materials, five legal cigarette brands from the US, UK and China, and three counterfeit samples. Regression lines are significant at p = 0.05, and slopes (m) are indicated (both pass through the origin within error).

The 14 samples analysed in this study span a greater range of arsenic concentrations than is normally encountered in cigarette tobacco [2] and cover a wide geographic range (US, EU, China) yet there is remarkable consistency in the fractions attributable to inorganic arsenic, MA and DMA species. Figure 2 shows how these vary with total sample As, with the slopes of the regression lines indicating that inorganic As is consistently present at about four times the sum of the measured organic species (MA + DMA), a ratio maintained in samples with greatly elevated levels (as in B6-B8 counterfeit products).

MA concentrations are generally lower (but not significantly) than those of DMA, or are below detectable limits. Overall the slopes of the regression lines indicate that approximately 80% of the arsenic species detected in these tobaccos is present as inorganic species with the remaining 20% as DMA and MA.

MA and DMA in plants is thought to be derived from soil rather than from *in planta* metabolism [29], as plants appear not to methylate inorganic As, unlike animals, bacteria and fungi [32], though methylated As species are readily translocated to the shoot once assimilated through the root system [26]. Low methylated As content in tobacco samples indicates low As methylation rates in the original growing environment. Relative concentrations of methylated species and total inorganic As species within the reference standards are generally stable, potentially due to growing environment conditions that favour inorganic As bioavailability to the plants (treatment with sewage-based nitrate or phosphate fertilisers), or application of arsenicals directly to the plant (perhaps atmospheric deposition of inorganic dust on leaves). As the presence of biological agents can affect the methylation of soil As, and thus passage into the food chain, as well as amount and source of the contaminant, soil properties, and the magnitude and rate of plant uptake and/or extent of absorption by animals [33] it is important to understand these factors in terms of As migration through both food chains and smoking in contributing to human exposure [34]. Soil has the capacity to buffer the effects of contaminants by binding these agents to soil constituents, or chemically converting them to inactive, insoluble or biologically unavailable forms. These factors alone make for complex dose response relationships in crops [34] and may provide a strategy for reducing the total As, and more specifically the inorganic As available to the plant, and therefore to the receptor.

### Implications for health

Human toxicity symptoms associated with exposure to arsenic include cancers of the lung and skin, and cardiovascular, gastrointestinal, hepatic and renal diseases [2,3]. Our findings on the prevalence of inorganic As species over organic forms, the former being the more toxic and persistent in the human body, could be directly relevant to gastrointestinal exposure during the use of oral tobacco. For smoking, taking the arsenic concentrations presented above we calculate that up to a few hundred ng per cigarette (based on 0.7 g cut tobacco in a single cigarette) could be transferred to the respiratory system under standard ISO smoking conditions using published transference factors [5]. The highest values are associated with the seized counterfeit products which are distributed illicitly, whereas legal products in the developed world would lead to less exposure. Smoking is a complex process and metal and metalloid components are not necessarily transferred unchanged on liberation from tobacco during the creation of smoke, New compounds may be more or less toxic than their precursor compounds, depending on redox conditions and other factors during combustion and smoke ageing. Such changes have recently been explored for arsenic. High resolution XANES spectra demonstrate the dominance of As(III) in smoke condensate [10] and HPLC-ICPMS analyses of smoke condensate indicate that inorganic species predominate [11]. These findings indicate that most of the arsenic to which the smoker is exposed is likely to be present in the more toxic species.

The comparative risks to smokers of exposure to individual hazardous smoke constituents, including metals and metalloids, can be modelled using the component’s concentration in typical smoke emissions, inhalation risks, consumption rates, and assumptions such as daily inhalation volume [6,35]. Assuming that the total risk is the sum of the risks of the individual components, the modelling approach has been applied to the comparison of different cigarette products [36]. Developing this further into a practical risk assessment tool is dependent on reliable analysis of metals and metalloids in smoke. Smoke analysis is analytically difficult and not widely practised compared with the analysis of tobacco. Widespread regulation based on the analysis of smoke emissions is a distant prospect but a methodology for comparing the risks of exposure to metals and metalloids in smoke using analyses of concentrations in precursor tobacco might provide a more practical way forward. This, however, is dependent on demonstrating that it is possible to make reliable predictions of exposure from analyses of tobacco and other parameters.

Tobacco is grown in over 120 countries [37]. It has long been known that arsenic concentrations in tobacco used to manufacture cigarettes varies with geographical region [5]. China and the United States are the largest producers of tobacco leaf in the developing and developed world respectively [37] and both are major consumers. A recent study estimated a mean value for arsenic of 0.29 mg kg^-1^ (standard deviation 0.04) in tobacco extracted from 50 samples of popular US cigarette brands [38], whereas the mean for 47 samples of popular cigarette brands in China is 0.85 mg kg^-1^ (standard deviation 0.73) [39]. These arsenic levels are significantly different (p < 0.001) suggesting that smokers in these countries may be exposed to very different levels of arsenic. If, as recent studies suggest, most of this arsenic will be present as an inorganic species and converted to reduced species on combustion, any risk of smoking-related disease due to arsenic exposure would appear to be considerably greater in China, a nation that is home to one quarter of the world’s smokers [40] who consumed 38% of the world’s cigarettes in 2009 [37].

### Implications for regulation

While the research literature includes numerous reports of heavy metal levels in tobacco [41] and a few studies of heavy metals in cigarette smoke [6-8] almost no attention has been paid to importance to toxicity of chemical and valence speciation. The lack of information on metal and metalloid speciation is largely due to the difficulties of detecting different species at the very low concentrations found in tobacco and smoke [12]. Such shortcomings are potentially exploitable by those opposing regulation.

If arsenic is to be regulated by means of its concentration in crops and commercial products, as has been recommended [2], then it will be necessary to demonstrate that (1) human exposure to arsenic in tobacco smoke can be predicted from its concentration in raw and processed tobacco, and (2) that a significant fraction of the arsenic to which the smoker is exposed is toxic or carcinogenic, i.e. essentially in the form of reduced inorganic arsenic species.

The dependence of arsenic concentration in smoke on its concentration in processed tobacco can be estimated. The tobacco in 50 popular US brands was analysed for metals and metalloids [38], and the same brands were smoked using the ISO and Health Canada intense smoking protocols with arsenic being analysed in the total particulate matter of mainstream smoke [9]. Analysis of the published data shows a significant positive correlation (r = 0.40, p < 0.01) between arsenic concentration in cigarette tobacco and its concentration in the total particulate matter of mainstream smoke generated under ISO conditions. The correlation was stronger (r = 0.59, p < 0.0001) for the same brands using the Health Canada intense smoking protocol, regarded by many as closer to real world smoking exposure [42]. A predictive model for the concentration of arsenic under a given smoking regimen needs take into account important factors including tobacco mass, length of cigarette rod and filter ventilation [9]. The range of arsenic in the US brands is narrow, approximately 0.2-0.4 μg g^-1^ [38] and any useful predictive model would need to span a much wider range reflecting global tobacco compositions. Notwithstanding, the US datasets demonstrate that such modelling is possible.

It is suggested here that the oxidation state of arsenic in tobacco leaf is irrelevant to the issue of regulation of products designed to be smoked. It has recently been shown that on combustion a cigarette liberates arsenic only in its As(III) oxidation state regardless of initial redox state in the precursor tobacco [10]. This is probably due to reduction processes in the burning zone and it is possible that As(III) is preferentially liberated. Furthermore, no evidence was found for oxidation to As(V) within 30 minutes of the conclusion of the smoking experiment. Note that an alternative view of the redox behaviour of arsenic has been presented [11] although the more recent study [10] had the advantage of the much greater sensitivity inherent in a third generation synchrotron.

These findings support the possibility of reducing the risks of smoking by regulation on the basis of arsenic concentration in tobacco crops and products assuming robust predictive models can be established for arsenic speciation and transference rates from leaf to smoke, as discussed above.

## Conclusions

The principal organic arsenic species detected in the 14 tobaccos analysed were DMA (detected in 13 samples) and MA (detected in 5 samples). Both organic and inorganic species increase with total arsenic concentration and regression analysis indicates that inorganic forms of arsenic dominate over all analysed organic species (DMA + MA) by a factor of 4:1 in tobaccos sampled over a wide range of compositions and geographical regions.

Given that inorganic arsenic is considered to be more toxic to humans than organic forms this has consequences for the health of users of oral tobacco and, depending on changes that may occur during combustion, also for the health of smokers.

A consequence of large regional differences in arsenic concentrations in tobacco may be geographical differences in degree of exposure and risk to health, China being notable for the high levels of arsenic in many of its cigarette brands.

The evidence presented in this paper provides tentative support for the recent proposal to regulate arsenic in tobacco crops and products [2] with the expectation that predictive models can be derived for exposure to inorganic arsenic species during smoking.
